# Norrin attenuates protease-mediated death of transformed retinal ganglion cells

**Published:** 2009-01-12

**Authors:** Song Lin, Mei Cheng, Wendelin Dailey, Kimberly Drenser, Shravan Chintala

**Affiliations:** 1 Eye Research Institute of Oakland University, Rochester, MI; 2 Department of Ophthalmology, William Beaumont Hospital, Royal Oak, MI

## Abstract

**Purpose:**

To investigate the effects of norrin, a nonconventional ligand for Wingless-Int (Wnt)-beta-catenin signaling pathway, on protease-mediated death of transformed rat retinal ganglion cells (RGC-5).

**Methods:**

Transformed RGC-5 cells were treated with 2.0 μM staurosporine (SS), a broad-spectrum protein kinase-C inhibitor, to induce growth arrest, differentiation, and elevated levels of tissue plasminogen activator (tPA) and urokinase plasminogen activator (uPA). RGC-5 cells were also treated with 2.0 μM SS and varying doses of recombinant norrin (3.125 to 100 ng/ml). Activation of Wnt pathway was assessed by nuclear translocation of beta-catenin. Proteolytic activity of tPA and uPA was determined by zymography assays and cell viability was determined by 3-[4,5-dimethylthiazol-2-yl]-2,5-diphenyltetrazolium bromide (MTT) assays. Expression and phosphorylation of the low-density lipoprotein-related receptor-1 (LRP-1), a cell surface receptor for tPA and uPA, was determined by immunoprecipitation and western blot analysis.

**Results:**

Compared to RGC-5 cells left untreated, cells treated with either SS alone or SS and norrin secreted elevated levels of tPA and uPA. A significant number of RGC-5 cells treated with only SS underwent cell death, whereas cells treated with SS and norrin did not, even though RGC-5 cells secreted elevated levels of tPA and uPA under both treatment conditions. Although norrin activated the Wnt pathway, Dickkopf related protein 1 (Dkk1), an inhibitor of Wnt/beta-catenin pathway, failed to completely block norrin’s neuroprotective effects. Assays for expression and phosphorylation of LRP-1 indicated that tPA and uPA cause RGC-5 cell death, in part, by reducing phosphorylation of LRP-1, whereas norrin attenuated tPA and uPA-mediated RGC cell death, in part, by restoring phosphorylation of LRP-1.

**Conclusions:**

Our results suggest that norrin attenuates tPA- and uPA-mediated death of RGC-5 cells by activating Wnt/beta-catenin pathway and by regulating phosphorylation of LRP-1.

## Introduction

Norrie disease, a severe and X-linked congenital retinal disorder, is characterized by aberrant vascularization, subretinal exudation, and retinal detachment [1]. The Norrie gene encodes a small, secreted, and cysteine-rich protein, termed norrin or Norrie disease protein (NDP) [2]. Mice that lack norrin have abnormal blood vessel growth in the vitreous and a disorganized retina [3]. In addition, mice with targeted disruption of NDP develop blindness due to lack of deep retinal capillaries, persistent hyaloid vessels, and growth of abnormal blood vessels in the vitreous [4,5]. Interestingly, transgenic expression of ectopic norrin in norrin-deficient *Ndp^y/-^* mice not only restores normal retinal vasculature, but also attenuates progressive loss of retinal ganglion cells (RGCs) [6]. Nonetheless, the mechanisms by which norrin attenuates loss of RGCs are unclear.

Recent studies have suggested that norrin acts as a ligand for Wingless–Int (Wnt) receptor-beta-catenin signal pathway, although norrin does not have sequence homology for the Wnt family of proteins [3]. Wnts, a family of approximately 20 secreted glycoproteins, initiate intracellular signal transduction by binding simultaneously to two cell surface receptors: a Frizzled (Fzd) receptor and a member of the low-density lipoprotein receptor-related protein (LRP) family, LRP-5 or LRP-6 [7,8]. The Frizzled receptors, seven-pass transmembrane receptors containing a cysteine-rich domain (CRD), act as binding site for Wnts, while the LRP-5 and LRP-6, single-pass transmembrane receptors, interact with both Fzd and Wnt [8]. An important difference between norrin and Wnts is that norrin activates Wnt/beta-catenin signal transduction pathway by specifically interacting with Frizzled-4 receptors, while Wnts can bind to multiple Frizzled receptors. The central player in Wnt pathways is a cytoplasmic protein, the beta-catenin, whose stability initiates the transcription of Wnt-target genes. When a Wnt is not bound to Fzd and LRP receptors, glycogen synthase kinase-3 (GSK-3) phosphorylates beta-catenin and targets it to degradation in the proteosomes. In contrast, Wnt binding to Fzd and LRP receptors inhibits activity of GSK-3; consequently, nonphosphorylated beta-catenin translocates to the nucleus where it forms complexes with members of T cell factor/lymphoid enhancer factor (TCE/LEF) members, and initiates the transcription of Wnt-target genes [8].

We have previously reported that elevated levels of two plasminogen activators, urokinase plasminogen activator (uPA) and tissue plasminogen activator (tPA), promote death of RGCs in vivo [9] and death of transformed retinal ganglion cells (RGC-5 cells) in vitro [10,11]. Here we report the effects of norrin on protease-mediated death of RGC-5 cells.

## Methods

### Materials

Dulbecco’s modified Eagle’s medium (DMEM), Dulbecco’s phosphate buffered saline (DPBS), penicillin, and streptomycin were obtained from Invitrogen Corporation (Carlsbad, CA). Staurosporine was obtained from Alexis Biochemicals (San Diego, CA). Human glu-plasminogen (product #410) and human fibrinogen (product #431) were obtained from American Diagnostica (Stamford, CT). Recombinant Dkk1 was obtained from R&D systems (Minneapolis, MN) and 3-[4,5-dimethylthiazol-2-yl]-2,5-diphenyltetrazolium bromide (MTT) was obtained from Sigma Chemical Company (St. Louis, MO).

### Cell culture

Transformed RGC-5 cells were cultured in DMEM containing 1 g/l glucose, 10% fetal bovine serum (FBS), 100 U/ml penicillin, and 100 μg/ml streptomycin. RGC-5 cells (from passage 10–20) were treated with 2.0 μM staurosporine to induce their differentiation as described previously [10,11]. Briefly, cells were cultured overnight in DMEM containing FBS. The next morning, cells were washed three times with phosphate buffered saline (PBS; 3.2 mM, Na_2_HPO_4_, 0.5 mM KH_2_PO_4_, 1.3 mM KCl, 135 mM NaCl, pH 7.4) and incubated in serum-free medium supplemented with 2.0 μM staurosporine. Where indicated, cells were also treated with SS+norrin, SS+Dkk1, and SS+H-89. Cells morphology was observed by using an inverted, phase contrast, and bright-field microscope, and digitized images were obtained by using a Nikon D100 digital camera (Nikon Corporation, Tokyo, Japan).

### Cell viability

Cells plated at 4×10^3^ cells/ml in 96 well tissue culture plates were left untreated or treated for 48 h with 2.0 μM SS along with indicated concentrations of norrin, Dkk1, and H-89 in serum-free DMEM. At 48 h after the treatment, cell viability was determined by incubating cells with 1.2 mM MTT for 2 h at 37 °C. At the end of 2 h, the formazan product produced by viable cells was dissolved in 0.01 M HCl, and the optical density was read at 570 nm by using an automated microplate reader (Bio-Tek Instruments, Inc., Winooski, VT). Statistical significance was analyzed by nonparametric Newman-Keuls analog procedure (GB Stat Software, Dynamic Microsystems, Silver Spring, MD).

### Activation of Wnt pathway

Accumulation of beta-catenin in the nucleus, an indicator for activation of Wnt pathway, was measured by western blot analysis [12]. Briefly, RGC-5 cells (2×10^6^) were plated in 100 mm tissue culture plates and incubated overnight at 37 °C. Afterwards, cells were washed three times with PBS and cells were either left untreated or treated for 24 h with 25 ng/ml norrin, SS (2.0 μM), or SS (2.0 μM) and norrin. At the end of treatment, cells were washed three times with PBS, scraped into 1 ml PBS, and transferred into individual Eppendorf tubes. Cells were centrifuged for 1 min at 16.1x g and the supernatants were discarded. Cells were then resuspended in 0.4 ml lysis buffer, composed of 10 mM HEPES, pH 7.9, 10 mM KCl, 0.1 mM EDTA, 0.1 mM EGTA, and 1 mM dithiothreitol, along with protease inhibitors (complete mini protease inhibitor cocktail, Roche Diagnostics). Cells were allowed to swell on ice for 20 min followed by the addition of 12.5 μl 10% Nonidet P-40. The tubes containing cells were vortexed vigorously for 10 s, and the homogenates were centrifuged at 16.1x g for 30 s. Supernatants containing cytoplasmic proteins were collected into fresh Eppendorf tubes. The nuclear pellets were resuspended in 25 μl ice-cold nuclear extraction buffer, composed of 20 mM HEPES, pH 7.9, 400 mM NaCl, 1 mM EDTA, 1 mM EGTA, 1 mM dithiothreitol, and protease inhibitors, then incubated on ice for 30 min with intermittent vortexing. Samples were centrifuged for 5 min at 4 °C (16.1x g), and the supernatants containing nuclear proteins were transferred into fresh Eppendorf tubes. Protein content was measured by using BCA protein assay reagent (Pierce, Rockford, IL) and aliquots containing equal amount of nuclear and cytoplasmic proteins (20 mg) were separated on 10% SDS-polyacrylamide gels. After separation, the proteins were transferred to polyvinylidene fluoride (PVDF) membranes and incubated for 1 h in 10% nonfat dry milk prepared in Tris-buffered saline containing 0.1% Tween-20 (TBS-T). PVDF membranes were incubated overnight with polyclonal antibodies against beta-catenin (Cell Signaling Technology, Danvers, MA). Membranes were washed with TBS-T and incubated for 1 h at room temperature with appropriate secondary antibodies conjugated to horseradish peroxidase (HRP). Finally, the proteins were detected by using an ECL chemiluminescence kit (Pierce, Rockford, IL). Equal loading of nuclear and cytoplasmic proteins was determined by re-probing the membranes with antibodies against TATA-binding protein (Abcam, Boston, MA) and actin, respectively. Relative level of beta-catenin was determined by using image analysis software (Scion Corporation, Frederick, MD), and the results were represented as mean arbitrary units. Statistical significance was analyzed by nonparametric Newman-Keuls analog procedure (GB Stat Software, Dynamic Microsystems).

### Phosphorylation of LRP-1

RGC-5 cells (2×10^6^ cells) plated in 100 mm tissue culture plates were either left untreated or were treated 24 h with 2.0 μM SS and indicated concentrations of norrin and H-89. At the end of 24 h, cells were washed twice with DPBS. Cells were scraped into 2 ml DPBS, transferred into pre-cooled 15 ml centrifuge tubes, and centrifuged at 13.4x g for 10 min at 4 °C. The supernatant was discarded and 500 μl of RadioImmuno Precipitation Assay (RIPA) buffer (1% nonidet-P40, 20 mM Tris-HCl, 150 mM NaCl, 1 mM Na_3_VO_4_, pH 7.4) buffer was added to each tube, and the tubes were incubated at 4 °C for 30 min on rotary shaker. Thirty min after incubation, cells were centrifuged and supernatants were collected into 1.5 ml Eppendorf tubes. Protein content was estimated by using Bio-Rad protein assay. Aliquots containing 500 μg total proteins from each treatment were incubated overnight 4 °C with 2 μl polyclonal antibodies against lipoprotein-related receptor-1 (LRP-1; Orbigen, San Diego, CA). Next, 100 μl Protein G-Sepharose (Invitrogen Corporation) was added to each sample, and samples were incubated at 4 °C for 4 h. Tubes containing Protein G-Sepharose were centrifuged for 2 min at 13.4x g and the supernatant was discarded. SDS-loading buffer (25 μl) was added to the pellet, and the tubes containing the pellet were boiled for 5 min. Tubes were centrifuged again to remove sepharose beads, and the supernatant was loaded onto 10% SDS–PAGE gels. After separation, the proteins were transferred to PVDF membranes and incubated for 1 h in 10% nonfat dry milk prepared in TBS-T. PVDF membranes were then probed with polyclonal antibodies against phosphoserine (Cell Signaling), phosphotyrosine (clone 4G10; Millipore, Billerica, MA), and 1:1,000 dilution LRP-1 (Orbigen). After overnight incubation with primary antibodies, membranes were washed twice with TBS-T and incubated for 1 h at room temperature with appropriate secondary antibodies conjugated to HRP. Finally, the proteins on the membranes were detected by using an enhanced chemiluminescence kit (Pierce). Relative level of proteins was determined by using Scion image analysis software, and the results from 3 independent experiments were represented as mean arbitrary units. Statistical significance was analyzed by non-parametric Newman-Keuls analog procedure (GB Stat Software, Dynamic Microsystems).

### Zymography

Proteolytic activity of tPA and uPA in RGC-5 cells was determined by plasminogen and fibrinogen zymography assays as previously described [10,11]. Briefly, aliquots containing 20 μl conditioned medium were mixed with 4X SDS gel-loading buffer and loaded onto 10% SDS polyacrylamide gels containing 5.5 mg/ml fibrinogen and 50 μg/ml plasminogen. After electrophoresis, the gels were washed three times with 2.5% Triton-X 100 (15 min each time) to remove SDS from the gels, and the gels were incubated overnight in 0.1 M glycine-buffer (pH 8.0) at 37 °C. After overnight incubation, the gels were stained with 0.2% Coomassie brilliant blue-R250 for 5 min and then destained with a solution containing 50% methanol and 10% acetic acid in deionized water. Proteolytic activity of tPA and uPA, evident as clear bands in the zymograms, was documented using the Kodak EDAS 290 imaging system (Eastman Kodak Company, Rochester, NY). Relative level of protease activity was determined by using image analysis software (Scion Corporation), and the results from 3 independent experiments were represented as mean arbitrary units. Statistical significance was analyzed by non-parametric Newman-Keuls analog procedure (GB Stat Software, Dynamic Microsystems).

## Results

### Effect of norrin on expression of tPA and uPA, and on death of RGC-5 cells

**Figure 1:**
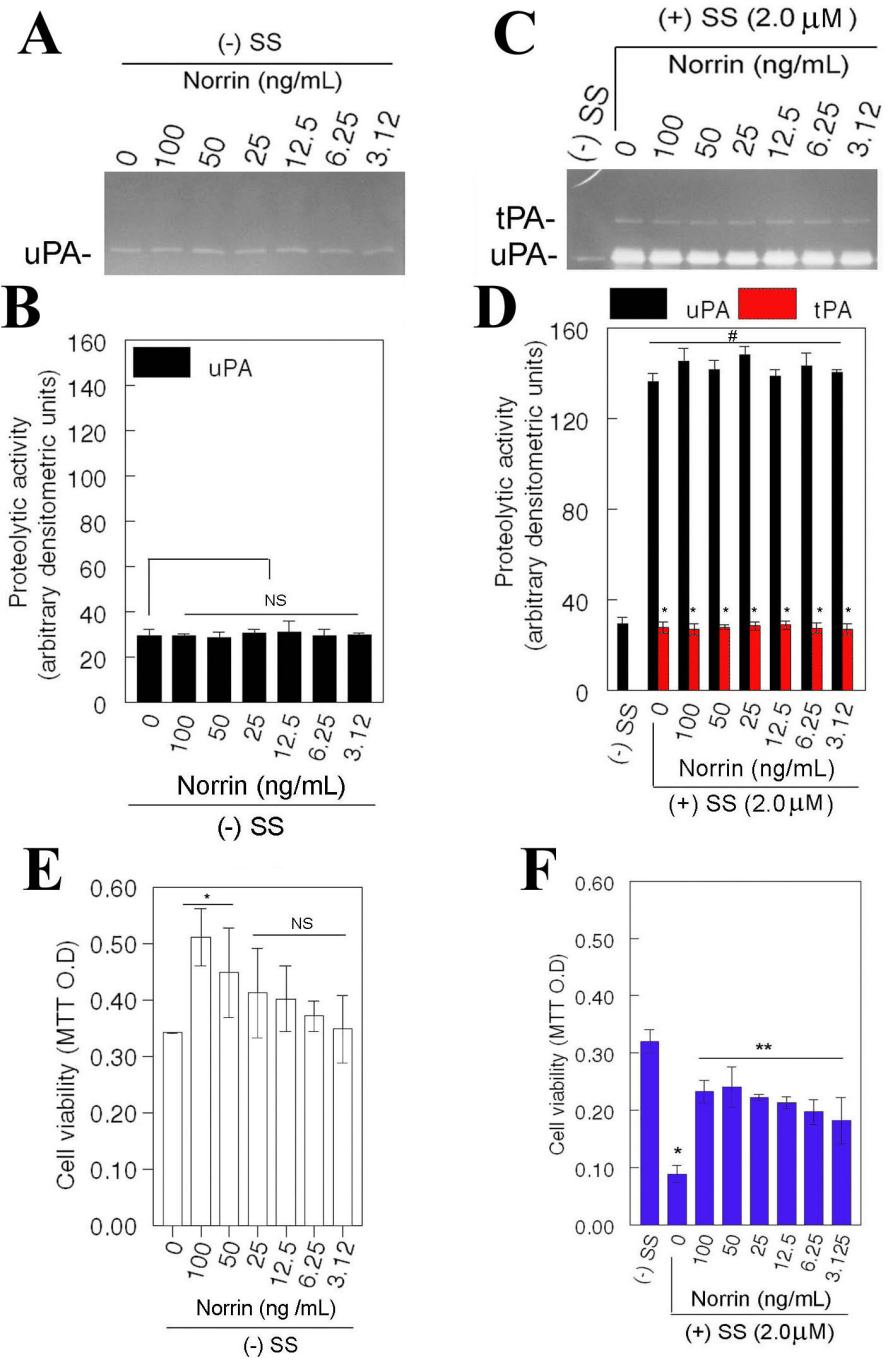
Effect of norrin on tPA and uPA expression, and on survival of RGC-5 cells. In the absence (**A,B,E**) or presence of 2.0 μM staurosporine (SS; **C,D,F**), RGC-5 cells were left untreated or treated for 48 h with varying concentrations of norrin (n=3 experiments). At the end of 48 h, conditioned medium was collected and proteolytic activity of tissue plasminogen activator (tPA) and and urokinase plasminogen activator (uPA) was determined by zymography assays (**A,C**), relative amount of proteolytic activity was determined by densitometric analysis (**B,D**), and cell viability was assessed by 3-[4,5-dimethylthiazol-2-yl]-2,5-diphenyltetrazolium bromide (MTT) assays (**E, F**). In the absence of SS, norrin had no effect on low levels of uPA expressed constitutively by RGC-5 cells (**A,B**; NS is not significant). In addition, lower concentrations of norrin had no effect on cell survival, but higher concentrations of norrin (50 and 100 ng/ml) increased cell survival significantly (**E**, *p<0.05). Compared to low levels of uPA expressed by untreated cells, uPA (**D**, #p<0.05) and tPA (**D**, **p<0.05) levels were significantly increased in SS alone or in SS and norrin-treated cells. Nonetheless, survival of RGC-5 cells decreased significantly in the presence of SS alone (**F**, *p<0.05), but not in the presence of SS and norrin (**F**, **p<0.05).

Throughout this study, RGC-5 cells were treated with 2.0 μM staurosporine to investigate the effects of norrin. This particular concentration was chosen because RGC-5 cells synthesize elevated levels of tPA and uPA at this concentration, and elevated levels of tPA and uPA, in turn, cause death of RGC-5 cells [10,11]. Cells were left untreated or treated for 48 h with varying concentrations of norrin alone, SS (2.0 μM) alone, or SS (2.0 μM) and varying concentrations of norrin. At the end of 48 h, conditioned medium was collected and proteolytic activity of tPA and uPA was determined by plasminogen/fibrinogen zymography assays. Zymography assays indicated that RGC-5 cells secreted low levels of uPA constitutively (Figure 1A). RGC-5 cells treated with increasing doses of norrin also secreted low levels of uPA similar to the levels secreted by untreated cells (Figure 1A,B) and the levels were not significantly different from untreated cells. In contrast, compared to untreated RGC-5 cells, cells treated with SS alone or SS and norrin secreted elevated levels of both uPA and tPA (Figure 1C), and the levels were significantly different from untreated cells (Figure 1C,D). Comparison of proteolytic activity (Figure 1B,D) with cell survival (Figure 1E,F) indicates that the survival of RGC-5 cells was significantly decreased under SS-treated conditions, but not under combined SS- and norrin-treated conditions despite the fact that RGC-5 cells synthesized similar levels of tPA and uPA under both treatment conditions.

**Figure 2:**
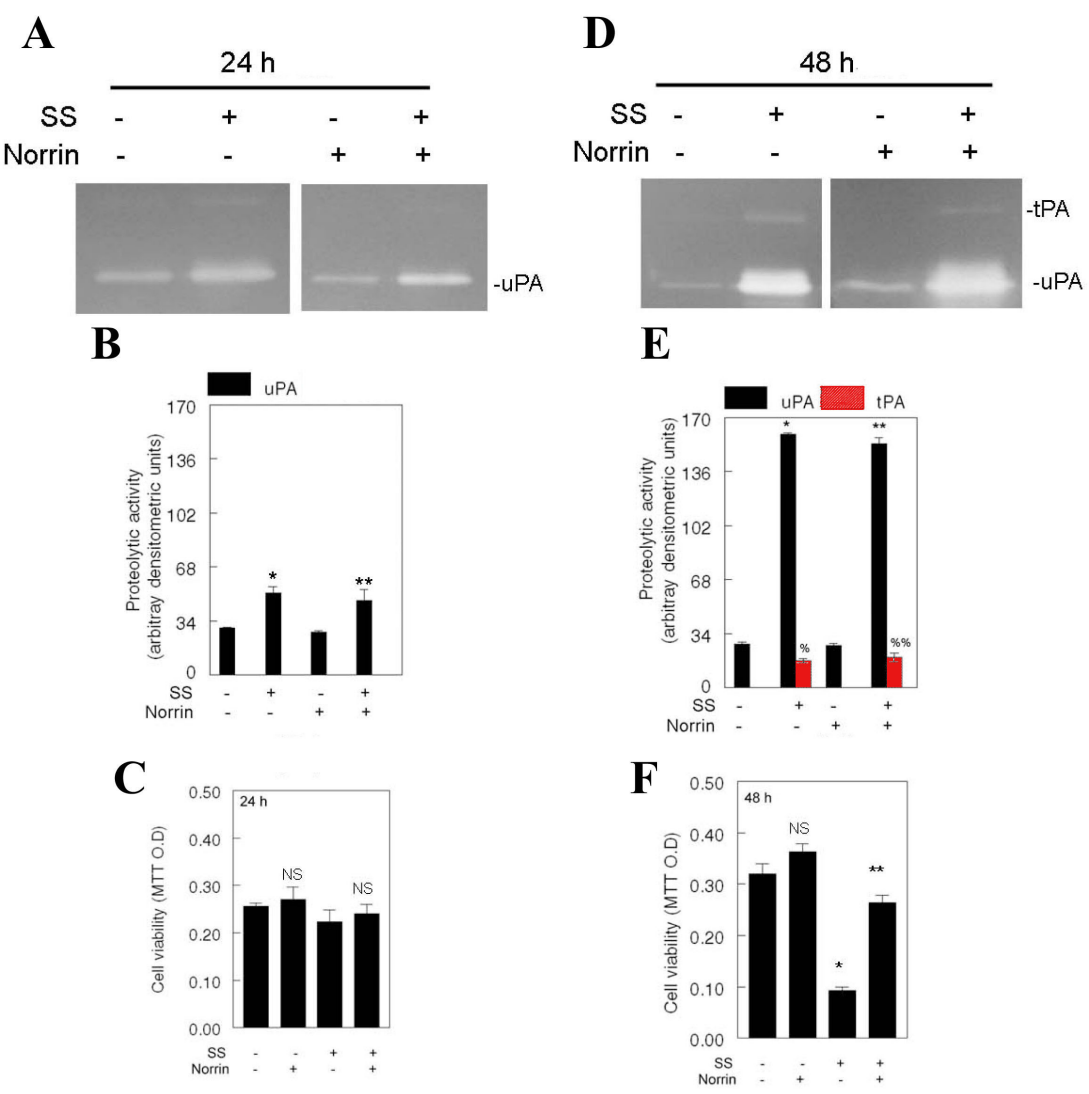
Time course effect of norrin on tPA and uPA expression, and on cell survival. RGC-5 cells were left untreated or treated for 24 h and 48 h with staurosporine (SS) only (2.0 μM), norrin only (25 ng/ml), or SS and norrin (n=3 experiments). At the end of the treatment, conditioned medium was collected, proteolytic activity of tissue plasminogen activator (tPA) and urokinase plasminogen activator (uPA) was determined by zymography assays (**A,D**), and relative amount of proteolytic activity was determined by densitometric analysis (**B,E**). Cell viability was determined by 3-[4,5-dimethylthiazol-2-yl]-2,5-diphenyltetrazolium bromide (MTT) assays (**C,F**). Compared to low levels of uPA present in untreated cells or in norrin alone-treated cells (**A,B**), uPA levels were increased in SS (**B**, *p<0.05) or in SS and norrin-treated cells (**B**, **p<0.05) at 24 h after treatment. However, increased levels of uPA alone had no effect on cell survival (**C**, not significant, NS). Compared to low levels of uPA present in untreated cells or in norrin alone-treated cells (**D,E**), uPA (**E**, *p<.0.05, **p<0.05) and tPA (**E**, %p<0.05, %%p<0.05) levels were increased significantly in SS or in SS and norrin-treated cells at 48 h after treatment. Nonetheless, survival of RGC-5 cells decreased in SS alone-treated cells (**F**, *p<0.05) but not in SS and norrin-treated cells (**F**,**p<0.05).

To investigate the time course effect of norrin on cell survival, we treated RGC-5 cells for 24 and 48 h with 2.0 μM SS with or without 25 ng/ml norrin. From this experiment forward, we chose 25 ng/ml norrin to treat the cells because norrin seems to elicit optimum effect on the expression of tPA and uPA, and on cell survival at this concentration (see Figure 1F). At the end of the treatment period, levels of tPA and uPA, and cell survival were investigated as described in the previous section. Zymography assays indicated that, regardless of the presence of norrin, RGC-5 cells synthesized similar levels of uPA at 24 h after treatment (Figure 2A,B) and both tPA and uPA at 48 h after treatment (Figure 2D,E). Cell viability assays indicated that low levels of uPA had no effect on cell survival at 24 h after treatment (Figure 2C). In contrast, increased levels of both tPA and uPA reduced the survival of RGC-5 cells at 48 h after treatment (Figure 2F). In addition, despite elevated levels of tPA and uPA, a significant number of RGC-5 cells survived in the presence of norrin (Figure 2F).

### Effect of norrin on Wnt-pathway activation

**Figure 3:**
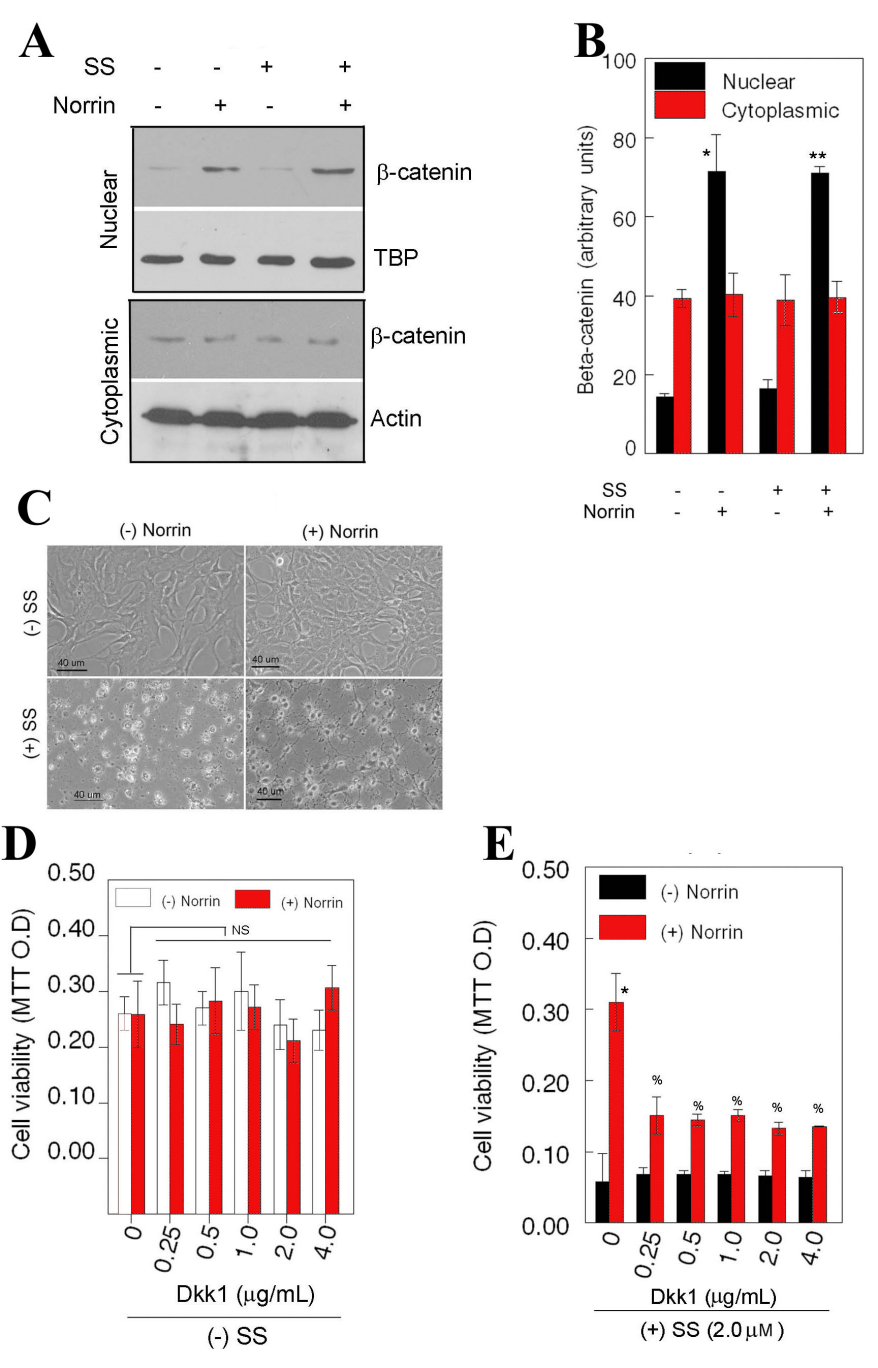
Effect of norrin on Wnt-beta-catenin pathway activation. Retinal ganglion cell (RGC)-5 cells were left untreated or treated for 24 h with 2.0 μM staurosporine (SS) only or 25 ng/ml SS and norrin. At the end of 24 h, cells were photographed (**C**), cytoplasmic and nuclear proteins were prepared (n=3 experiments), and aliquots containing equal amount of protein (20 μg) were subjected to western blot analysis by using antibodies against beta-catenin (**A**). Equal loading of nuclear and cytoplasmic proteins was determined by reprobing the membranes with antibodies against TATA binding protein (TBP) and actin, respectively (**A**). Compared to untreated cells, beta-catenin levels in nuclear fractions were increased significantly in norrin-treated cells (**A,B**, *p<0.05) and in SS and norrin-treated cells (**A,B**,**p<0.05). Micrographs of cells (**C**) indicate that elevated levels of beta-catenin associated with increased cell survival. To determine whether inhibition of Wnt pathway reduces norrin-mediated cell survival, cells were left untreated or treated for 48 h with norrin (25 ng/ml), Dkk1 (0.25-4.0 μg/ml), or Dkk1 and norrin, in the absence (**D**) or presence of 2 μM SS (**E**) and cell viability was assessed by 3-[4,5-dimethylthiazol-2-yl]-2,5-diphenyltetrazolium bromide (MTT) assays (n=3 experiments). In the absence SS, Dkk1 had no effect on cell survival regardless of the presence of norrin (**D**, NS is not significant). Compared to SS-treated cells, survival of SS and norrin-treated cells increased significantly (**E**,*p<0.05). Although Dkk1 reduced norrin-mediated cell survival of RGC-5 cells (**E**, %p<0.05), Dkk1 failed to reduce the survival similar to that observed under SS alone-treated conditions.

To investigate whether Wnt pathway plays a role in norrin-mediated protective effect, we employed western blot analysis to determine the translocation of beta-catenin into the nucleus. Western blot analysis performed on nuclear extracts prepared from RGC-5 cells indicated that a low level of beta-catenin was constitutively present in nuclear proteins extracted from cells left untreated or treated with norrin (Figure 3A). In contrast, in the presence of norrin, a significant increase in beta-catenin levels was observed in nuclear proteins extracted from cells treated with norrin or both SS and norrin (Figure 3A,B) indicating that norrin, indeed, activates Wnt-beta-catenin pathway. Comparison of nuclear translocation of beta-catenin with cell viability (Figure 3C) indicated that increased survival of RGC-5 cells under combined norrin- and SS-treated conditions associated with nuclear translocation of beta-catenin.

To determine whether inhibition of Wnt-beta-catenin pathway reduces norrin's protective effect, RGC-5 cells were left untreated or treated with norrin, Dkk1 (a potent inhibitor for Wnt-beta-catenin pathway; 0.25-4.0 μg/ml) [13] or Dkk1 and norrin. In addition, cells were treated with either SS only, SS and norrin, SS and Dkk1, or combined SS, norrin, and Dkk1. Results presented in Figure 3D indicate that in the absence of SS, Dkk1 had no effect on survival of RGC-5 cells regardless of norrin’s presence. In contrast, in the presence of SS, norrin enhanced survival of RGC-5 cells (Figure 3E), consistent with results presented in Figure 1F. When cell survival under combined SS- and norrin-treated conditions was compared with combined SS-, norrin-, and Dkk1-treatment, although cell survival was reduced to a large extent with Dkk1, cell survival was not reduced to a level observed under SS alone-treated conditions (Figure 3E). These results suggest that beta-catenin activation alone is insufficient to account for norrin-mediated protective effect on RGC-5 cells.

### Effect of norrin on phosphorylation of LRP-1

To investigate the mechanisms (other than Wnt/beta-catenin pathway) by which norrin attenuated tPA and uPA-mediated death of RGC-5 cells, we have focused our attention on LRP-1 because tPA and uPA induces death of RGC-5 cells by interacting with this cell surface receptor. First, we investigated whether norrin downregulates the expression of LRP-1. Our western blot studies indicated that RGC-5 cells left untreated or treated with either SS only or combined SS and norrin expressed similar levels of LRP-1 protein (data not shown). These results indicate that downregulation of LRP-1 is not responsible for norrin-mediated survival of RGC-5 cells.

**Figure 4:**
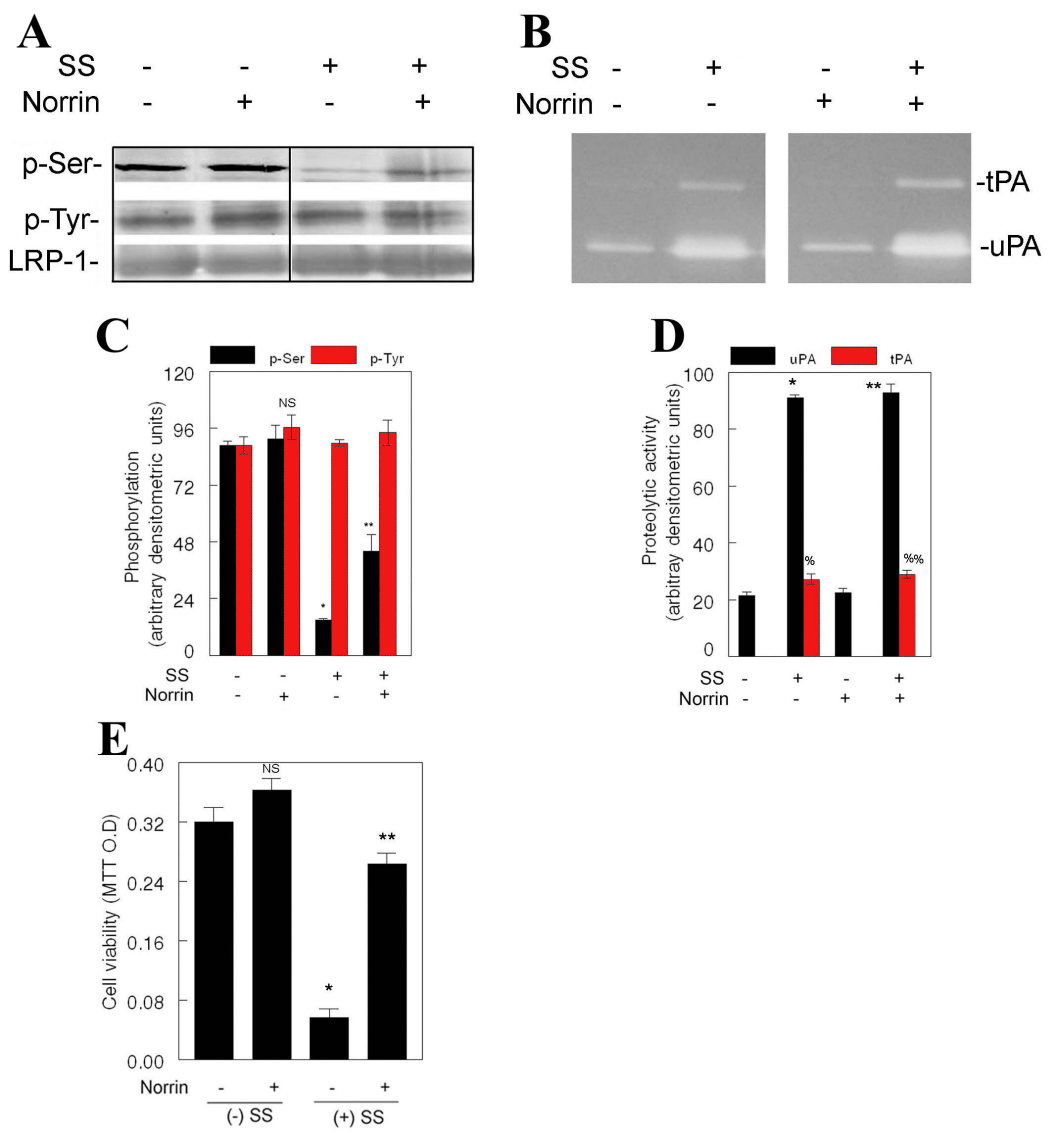
Effect of norrin on LRP-1 phosphorylation. Retinal ganglion cell (RGC)-5 cells were left untreated or treated for 24 h with 2.0 μM staurosporine (SS) alone or SS and 25 ng/ml norrin (n=2 experiments). At the end of 24 h, cells were collected, proteins were extracted, and immunoprecipitated by using antibodies against lipoprotein-related receptor-1 (LRP-1). Immunoprecipitated proteins were subjected to western blot analysis by using antibodies against anti-phosphoserine, anti-phosphotyrosine, and anti-LRP-1 (**A**). Relative levels of proteins were determined by densitometry (**C**). At the end of treatment, conditioned medium was collected, and proteolytic activity of tissue plasminogen activator (tPA) and urokinase plasminogen activator (uPA) was determined by zymography assays (**B**). Relative amount of proteolytic activity was determined by densitomety (**D**). LRP-1 was expressed constitutively in untreated cells and its expression did not change regardless of the treatment condition (**A**). LRP-1 was phosphorylated at Tyr-residues constitutively in untreated cells and phosphorylation status of LRP-1 at Tyr-residuces was not altered with any of the treatment conditions (**A**). LPR-1 was constitutively phosphorylated at Ser-residues in untreated cells (**A**). Norrin-treatment alone had no effect on LPR-1 phosphorylation at Ser-residues, but SS-treatment significantly reduced Ser-phosphorylation of LRP-1 (**A, C**, *p<0.05). Nonetheless, LPR-1 phosphorylation at Ser-residues was significantly increased in the presence of SS and norrin (**A,C**, **p<0.05). RGC-5 cells were left untreated or treated for 48 h with 2.0 μM SS and 25 ng/ml norrin in serum-free medium (n=3 experiments). Compared to low levels of uPA in untreated cells, levels of both uPA (**B,D**, *p<0.05) and tPA (**B,D**, %p<0.05) were increased in the presence of SS. In addition, compared to low levels of uPA in norrin-treated cells, levels of both uPA (**B,D**, **p<0.05) and tPA (**B,D**, %%p<0.05) were significantly increased in the presence of SS and norrin. Nonetheless, cell viability assays (**E**) indicate that survival of RGC-5 cells decreased significantly under SS-treated conditions (**E**,*p<0.05), but not under SS and norrin-treated conditions (**E**, **p<0.05). NS, not significant.

Since recent studies indicated that phosphorylation status of the cytoplasmic domain of LRP-1 plays a crucial role in signal transduction [14-16], we have hypothesized that phosphorylation status of LRP-1 may underlie norrin-mediated survival of RGC-5 cells. To investigate this hypothesis, RGC-5 cells were left untreated or treated with norrin alone, SS alone, or SS and norrin. At the end of the treatment, proteins were extracted and immunoprecipitated by using antibodies against LRP-1. Immunoprecipitated protein complexes were then subjected to western blot analysis by using antibodies against pSer and pTyr residues. Western blot analysis indicated constitutive phosphorylation of LRP-1 at both Ser and Tyr-residues in cells left untreated or treated with norrin (Figure 4A,B). When cells were treated with SS, LRP-1 phosphorylation was reduced at Ser-residues, but not at Tyr-residues (Figure 4A,B). In contrast, Ser-phosphorylation of LRP-1, reduced by SS, was increased largely when cells were treated with SS and norrin (Figure 4A,B). These results indicate that norrin attenuates the effect of SS, in part, by regulating the phosphorylation of LRP-1. Comparison of phosphorylation of LRP-1 with tPA and uPA levels (Figure 4C,D) indicated that the phosphorylation status of LRP-1 does not affect the levels of tPA and uPA. However, comparison of phosphorylation of LRP-1 (Figure 4A) with MTT assays (Figure 4E) indicated that phosphorylation status of LRP-1 affected the survival of RGC-5 cells. Results presented in Figure 4 show that despite elevated levels of tPA and uPA (Figure 4C,D), a significant number of RGC-5 cells survived (Figure 4E) as long as LRP-1 was phosphorylated at both Ser and Tyr-residues (Figure 4A).

**Figure 5:**
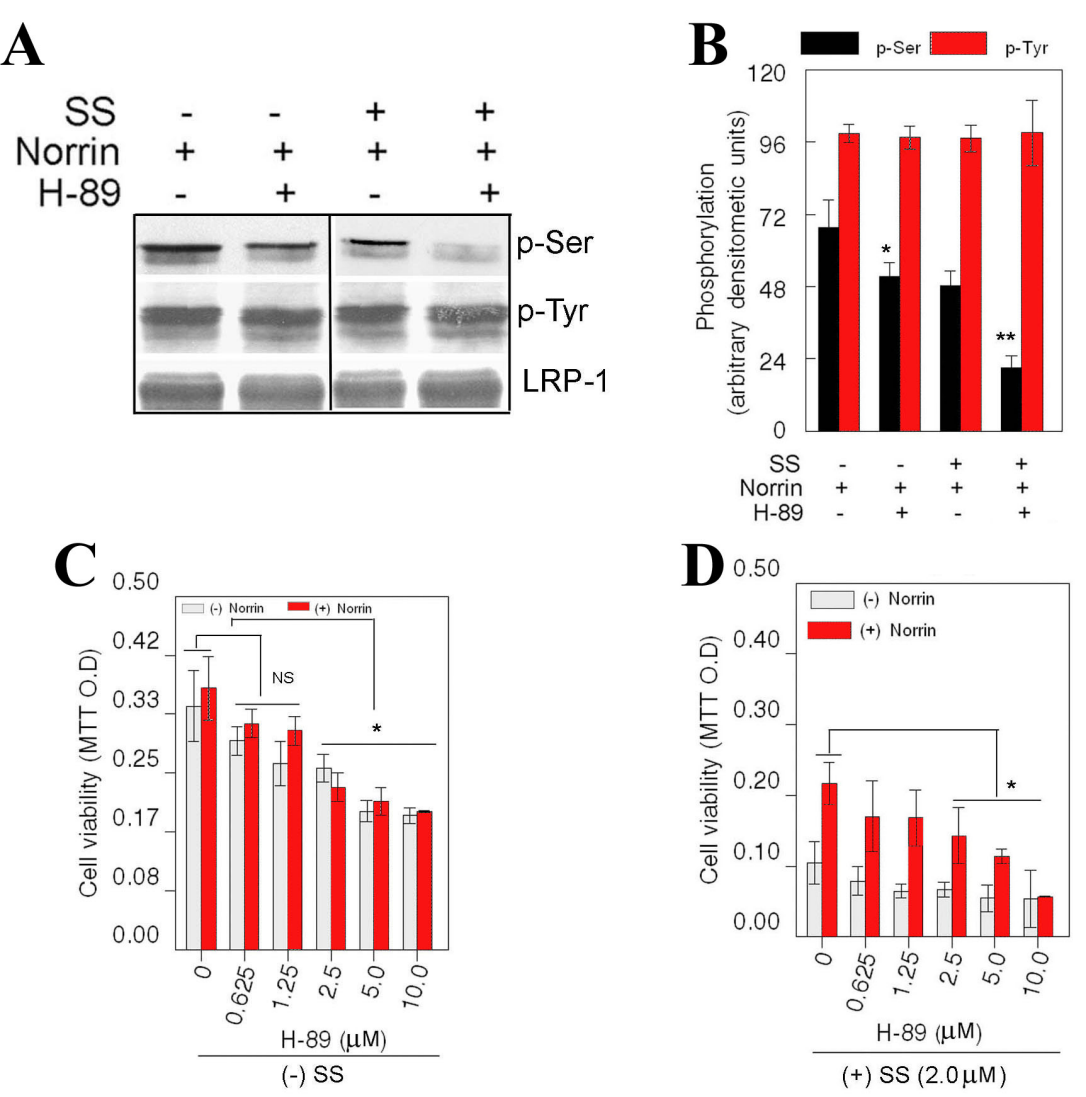
Effect of protein kinase inhibitors on phosphorylation of LRP-1. Retinal ganglion cell (RGC)-5 cells were left untreated or treated for 24 h with staurosporine (SS; 2.0 μM), norrin (25 ng/ml), with or without H-89 (n=3 experiments). At the end of 24 h, proteins were extracted and immunoprecipitated by using antibodies against lipoprotein-related receptor-1 (LRP-1). Immunoprecipitated proteins were then subjected to western blot analysis by using antibodies against phosphoserine, phosphotyrosine, and LRP-1 (**A**). Relative amount of proteins was determined by densitometric analysis (**B**). LRP-1 was expressed constitutively in norrin-treated cells and its expression did not change regardless of treatment condition (**A**). LRP-1 was phosphorylated at Tyr-residues constitutively in norrin-treated cells and phosphorylation status of LRP-1 at Tyr-residues did not change with any of the treatment condition (**A**). In addition, LPR-1 was constitutively phosphorylated at Ser-residues in norrin-treated cells (**A**). Compared to Ser-phosphorylation of LRP-1 in norrin-treated cells, Ser-phosphorylation of LRP-1 was significantly reduced when cells were treated with H-89 (**A, B**, *p<0.05). In addition, compared to Ser-phosphorylation of LRP-1 under SS and norrin-treated conditions, Ser-phosphorylation of LRP-1 was further reduced under H-89, SS, and norrin-treated conditions (**A,B**, **p<0.05). Cell viability assays indicate that, in the absence of SS, lower concentrations of H-89 had no effect on cell survival, but higher concentrations of H-89 (2.5–10.0 μM) decreased cell survival significantly regardless of norrin's presence (**C**, *p<0.05). Furthermore, in the presence of SS, lower concentrations of H-89 had no effect on cell survival, but higher concentrations of H-89 (2.5–10.0 μM) decreased norrin-mediated cell survival (**D**, *p<0.05).

To identify the kinases responsible for LRP-1 phosphorylation, we have concentrated our efforts on protein kinase A (PKA) and protein kinase C (PKC) because these kinases have been shown to regulate the phosphorylation of LRP-1. Since SS (inhibitor for PKCs) reduced the phosphorylation of LRP-1 at Ser-residues in RGC-5 cells (see Figure 4A), we have ruled out the role of PKCs in LRP-1 phosphorylation under SS and norrin-treated conditions. To investigate whether PKA plays a role in norrin-mediated phosphorylation of LRP-1, RGC-5 cells were left untreated or treated with 5 μM H-89 (a PKA inhibitor) [16], norrin alone, or combined norrin and 5 μM H-89. In addition, RGC-5 cells were treated with SS only, SS and H-89, SS and norrin, or combined SS, norrin, and H-89. After the treatment, phosphorylation of LRP-1 was assessed by western blot analysis as described in the previous section. Western blot analysis indicated that LRP-1 was phosphorylated at Ser- and Tyr-residues when treated with norrin alone or norrin plus SS (Figure 5A,B), consistent with the results presented in Figure 4A. When LRP-1 phosphorylation was compared between norrin only or norrin and H-89-treated cells, Ser-phosphorylation of LRP-1 was reduced by about 15%–20%. In the presence of SS and norrin, H-89 led to an additional reduction in LRP-1 phosphorylation, suggesting that PKA plays a role in norrin-mediated phosphorylation of LRP-1.

Finally, to investigate the role of PKA on survival of RGC-5 cells, cells were left untreated or treated with 0.625–10 μM H-89 only, norrin only, or combined norrin and 0.625–10 μM H-89, with or without SS. At the end of the treatment cell viability was determined by MTT assays (Figure 5C,D). Results presented in Figure 5C indicate that 2.5−10 μM H-89 alone, in the presence or absence of norrin, significantly reduced the viability of RGC-5 cells, indicating that PKA, in part, plays a role in survival of RGC-5 cells under normal conditions. In addition, when survival of RGC-5 cells was compared between SS- and norrin-treated conditions, and combined SS-, norrin-, and H-89-treated conditions (Figure 5D), cell survival was further reduced under 2.5–10 μM H-89-treated conditions. These results indicate that PKA, in part, plays a role in norrin-mediated survival of RGC-5 cells.

## Discussion

Recent studies indicated that norrin, which regulates the regression of hyaloid vessels in the retina [3,6,7,17,18] and acts as a nonconventional ligand for Wnt pathway, prevents loss of RGCs in norrin-deficient *Ndp^y^/-* mice [6]. Yet, it is unclear how norrin attenuates loss of RGCs.

In this study, we have used transformed RGC-5 cells as an in vitro model system to investigate the effects of norrin on cell death. We have chosen RGC-5 cells because they express markers such as Thy-1, Brn-3c, and neuritin characteristic of primary RGCs, and have extensively been used as an in vitro model system to investigate the effect of oxidative stress [19], sigma-1 receptor ligands [20], thioredoxins [21], visible light exposure [22], and hydrostatic pressure [23,24]. Although RGC-5 cells have been widely used, they differ from primary RGCs in many respects including their proliferative nature and resemblance to glial cells in culture. Studies by Frassetto et al. [25] have reported that RGC-5 cells can be differentiated into neuronal-like cells by treating them with SS, a broad-spectrum protein kinase inhibitor. By employing RGC-5 cells and by treating them with SS, we have previously reported that SS induces tPA and uPA expression in RGC-5 cells, and elevated levels of these proteases directly cause the death of RGC-5 cells by interacting with LRP-1 [10,11]. Therefore, we have used RGC-5 cells to investigate the effect of norrin on tPA and uPA-mediated cell death, and made several important observations: 1) norrin attenuated SS-mediated death of RGC-5 cells, without altering the levels of tPA and uPA; 2) norrin-mediated survival of RGC-5 cells was associated with activation of Wnt/beta-catenin pathway, but inhibition of Wnt pathway did not reduce norrin-mediated survival of RGC-5 cells completely; and 3) norrin attenuated tPA and uPA-mediated death of RGC-5 cells, in part, by regulating the phosphorylation status of LRP-1, a cell surface receptor for both tPA and uPA.

**Figure 6:**
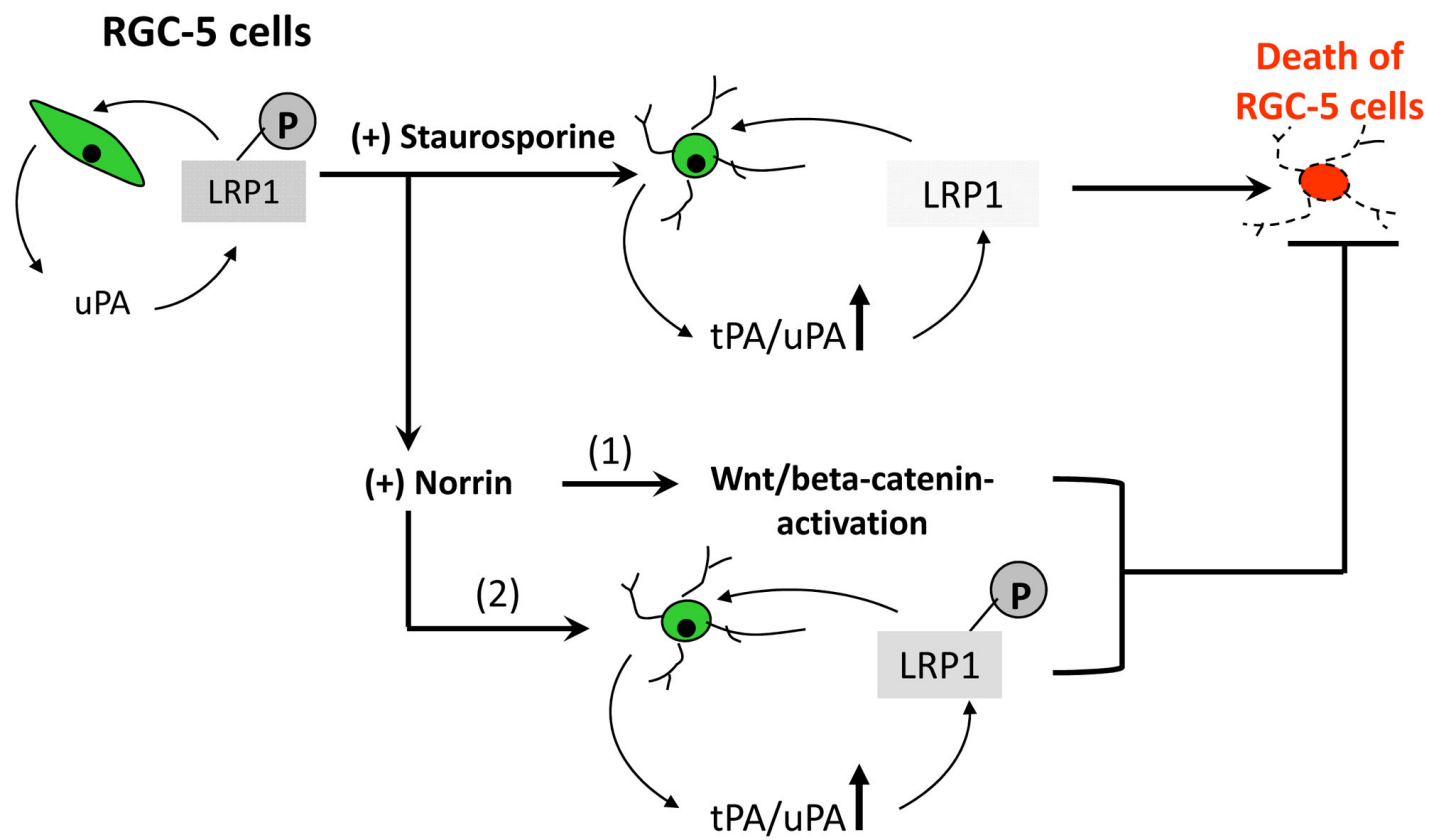
Proposed model for norrin’s role on RGC-5 cells. Our working hypothesis is that under normal conditions, phosphorylated LRP-1 receptor prevents uPA-mediated cell death. SS, by inhibiting PKC, downregulates LRP-1 phosphorylation, and in the absence of LRP-1 phosphorylation, elevated levels of tPA and uPA cause death of RGC-5 cells. In contrast, norrin prevents tPA and uPA-mediated cell death, in part, by restoring phosphorylation of LRP-1 and, in part, by activating Wnt pathway.

Based on the results presented in this study, we propose that both PKA and PKC contribute to constitutive phosphorylation of LRP-1 in undifferentiated RGC-5 cells (Figure 6). Under these conditions, low levels of uPA do not cause death of RGC-5 cells because LRP-1 is phosphorylated constitutively. Since SS is a PKC inhibitor, we propose that SS reduces PKC levels in differentiated cells, and the reduced PKC fails to phosphorylate LRP-1. We believe that under these conditions, elevated levels of tPA and uPA cause death of RGC-5 cells because LRP-1 is not phosphorylated at Ser-residues. On the other hand, with addition of norrin, LRP-1 remains phosphorylated at the Ser-residues, and under these conditions, survival of RGC-5 cells increases despite elevated levels of tPA and uPA. We propose that Wnt pathway alone is not responsible for the norrin-mediated protective effect since inhibition of Wnt pathway did not decrease norrin-mediated protection completely. Since norrin restored LRP-1 phosphorylation and attenuated cell death despite elevated levels of tPA and uPA, and since norrin-mediated protection was reduced by the PKA inhibitor, we propose that norrin restores phosphorylation of LRP-1, in part, by regulating PKA levels in RGC-5 cells. Finally, we propose that as long as LRP-1 is phosphorylated, elevated levels of tPA and uPA do not cause death of RGC-5 cells.

It is interesting to speculate the mechanisms underlying phosphorylation of LRP-1 and survival of RGC-5 cells. Although a growing body of evidence indicates that LRP-1 plays a role in the pathophysiology of cerebral ischemia [26-29] and deletion of LRP-1 in mouse is embryonic lethal [30], two important aspects of LRP-1 are still unclear. First, how does LRP-1 regulates cell survival? Second, does phosphorylation status of LRP-1 receptor plays a role in cell survival? Since the cytoplasmic domain of LRP-1 contains NPXY motifs that bind to a variety of adaptor proteins, it has been speculated that LRP-1 controls a variety of cells functions including cell survival [31]. For example, previous studies have suggested that LRP-1 may regulate cell survival by activating the PI3-Akt pathway [32,33], by preventing nuclear translocation of activated c-Jun N-terminal protein kinase (JNK) to the nucleus [34] or by controlling activation of caspases [32]. In addition, several previous studies have used the receptor-associated protein (RAP), a LRP-1 receptor antagonist, and reported that LRP-1 regulates several cellular functions [35-38]. Furthermore, studies from our own laboratory have reported that RAP inhibits tPA and uPA interaction with LRP-1, and attenuates tPA and uPA-mediated death of RGC-5 cells [11]. However, the link between phosphorylation status of LRP-1 and cell death has not been reported. To our knowledge, this is the first report describing the link between phosphorylation of LRP-1 and cell death, at least in transformed RGC-5 cells.

The precise mechanisms by which norrin affects the phosphorylation of LRP-1 are currently unclear. It is plausible that norrin may regulate certain kinases and the kinases, in turn, may alter the phosphorylation of LRP-1. In this context, previous studies have shown that the signaling functions of LRP-1 are mediated through phosphorylation of its cytoplasmic domain [4,39]. For example, PKA plays a role in Ser-phosphorylation [14], while platelet-derived growth factor (PDGF) induces phosphorylation of Tyr in the cytoplasmic domain of LRP-1 [15]. In our study, we have shown that norrin regulates phosphorylation of LRP-1 through PKA. An additional possibility is that certain genes activated by Wnt pathway may, in turn, regulate norrin-mediated protective effect in RGC-5 cells. Finally, LRP-1 can alter the Wnt pathway by disrupting LRP5/6 and Frizzled complexes [40].

There are few caveats associated with study. First, the current study used transformed RGC-5 cells for investigating the effect of norrin. Therefore, the results presented in this study are applicable only to cultured RGC-5 cells, and not applicable to primary RGCs. Second, since the current used SS (a PKC inhibitor) to differentiate RGC-5 cells and to induce death of RGC-5 cells, the relevance of these results to a clinical situation is a concern. Yet, based on recent studies that used SS to induce death of RGCs in animal models, we believe that these results will also be applicable to clinical situations. For example, Cordeiro et al. [41] have injected SS into the vitreous humor of dark Agouti rats and macaque monkeys to induce apoptosis of RGCs, and reported that this model system is useful to investigate the mechanisms underlying retinal neuro-degenerative diseases. Furthermore, recent studies indicate that the SS-induced model system represents as a glaucoma-related animal model to investigate the role of glutamate receptors in RGCs’ death [42]. Future studies aimed at understanding the effect of norrin in animal models of SS-induced cell death would provide a better understanding of the mechanisms underlying norrin in survival of RGCs.

In summary, we have provided evidence that norrin attenuates tPA and uPA-mediated death of RGC-5 cells by activating Wnt/beta-catenin pathway and by regulating the phosphorylation of LRP-1, a cell surface receptor for both tPA and uPA.

## References

[1] Berger W, Ropers HH. Norrie disease. In: Scrivers CR, Beaudet AL, Sly WS, Valle D, editors. The metabolic and molecular bases of inherited disease. 8th ed. New York: McGraw Hill; 2001. p. 5977–85.

[2] Meitinger T, Meindl A, Bork P, Rost B, Sander C, Haasemann M, Murken J (1993). Molecular modelling of the Norrie disease protein predicts a cystine knot growth factor tertiary structure.. Nat Genet.

[3] Xu Q, Wang Y, Dabdoub A, Smallwood PM, Williams J, Woods C, Kelley MW, Jiang L, Tasman W, Zhang K, Nathans J (2004). Vascular development in the retina and inner ear: control by Norrin and Frizzled-4, a high-affinity ligand-receptor pair.. Cell.

[4] Berger W, van de Pol D, Warburg M, Gal A, Bleeker-Wagemakers L, de Silva H, Meindl A, Meitinger T, Cremers F, Ropers HH (1992). Mutations in the candidate gene for Norrie disease.. Hum Mol Genet.

[5] Berger W (1998). Molecular dissection of Norrie disease.. Acta Anat (Basel).

[6] Ohlmann A, Scholz M, Goldwich A, Chauhan BK, Hudl K, Ohlmann AV, Zrenner E, Berger W, Cvekl A, Seeliger MW, Tamm ER (2005). Ectopic norrin induces growth of ocular capillaries and restores normal retinal angiogenesis in Norrie disease mutant mice.. J Neurosci.

[7] Masckauchan TN, Kitajewski J (2006). Wnt/Frizzled signaling in the vasculature: new angiogenic factors in sight.. Physiology (Bethesda).

[8] Logan CY, Nusse R (2004). The Wnt signaling pathway in development and disease.. Annu Rev Cell Dev Biol.

[9] Mali RS, Cheng M, Chintala SK (2005). Plasminogen activators promote excitotoxicity-induced retinal damage.. FASEB J.

[10] Harvey R, Chintala SK (2007). Inhibition of plasminogen activators attenuates the death of differentiated retinal ganglion cells and stabilizes their neurite network in vitro.. Invest Ophthalmol Vis Sci.

[11] Rock N, Chintala SK (2008). Mechanisms regulating plasminogen activators in transformed retinal ganglion cells.. Exp Eye Res.

[12] Jian H, Shen X, Liu I, Semenov M, He X, Wang XF (2006). Smad3-dependent nuclear translocation of beta-catenin is required for TGF-beta1-induced proliferation of bone marrow-derived adult human mesenchymal stem cells.. Genes Dev.

[13] Zorn AM (2001). Wnt signalling: antagonistic Dickkopfs.. Curr Biol.

[14] Li Y, van Kerkhof P, Marzolo MP, Strous GJ, Bu G (2001). Identification of a major cyclic AMP-dependent protein kinase A phosphorylation site within the cytoplasmic tail of the low-density lipoprotein receptor-related protein: implication for receptor-mediated endocytosis.. Mol Cell Biol.

[15] Loukinova E, Ranganathan S, Kuznetsov S, Gorlatova N, Migliorini MM, Loukinov D, Ulery PG, Mikhailenko I, Lawrence DA, Strickland DK (2002). Platelet-derived growth factor (PDGF)-induced tyrosine phosphorylation of the low density lipoprotein receptor-related protein (LRP). Evidence for integrated co-receptor function betwenn LRP and the PDGF.. J Biol Chem.

[16] Amos S, Mut M, diPierro CG, Carpenter JE, Xiao A, Kohutek ZA, Redpath GT, Zhao Y, Wang J, Shaffrey ME, Hussaini IM (2007). Protein kinase C-alpha-mediated regulation of low-density lipoprotein receptor related protein and urokinase increases astrocytoma invasion.. Cancer Res.

[17] Clevers H (2004). Wnt signaling: Ig-norrin the dogma.. Curr Biol.

[18] Drenser KA, Fecko A, Dailey W, Trese MT (2007). A characteristic phenotypic retinal appearance in Norrie disease.. Retina.

[19] Nakajima Y, Inokuchi Y, Nishi M, Shimazawa M, Otsubo K, Hara H (2008). Coenzyme Q(10) protects retinal cells against oxidative stress in vitro and in vivo.. Brain Res.

[20] Tchedre KT, Yorio T (2008). sigma-1 receptors protect RGC-5 cells from apoptosis by regulating intracellular calcium, Bax levels, and caspase-3 activation.. Invest Ophthalmol Vis Sci.

[21] Munemasa Y, Kim SH, Ahn JH, Kwong JM, Caprioli J, Piri N (2008). Protective effect of thioredoxins 1 and 2 in retinal ganglion cells after optic nerve transection and oxidative stress.. Invest Ophthalmol Vis Sci.

[22] Wood JP, Lascaratos G, Bron AJ, Osborne NN (2008). The influence of visible light exposure on cultured RGC-5 cells.. Mol Vis.

[23] Liu Q, Ju WK, Crowston JG, Xie F, Perry G, Smith MA, Lindsey JD, Weinreb RN (2007). Oxidative stress is an early event in hydrostatic pressure induced retinal ganglion cell damage.. Invest Ophthalmol Vis Sci.

[24] Ju WK, Liu Q, Kim KY, Crowston JG, Lindsey JD, Agarwal N, Ellisman MH, Perkins GA, Weinreb RN (2007). Elevated hydrostatic pressure triggers mitochondrial fission and decreases cellular ATP in differentiated RGC-5 cells.. Invest Ophthalmol Vis Sci.

[25] Frassetto LJ, Schlieve CR, Lieven CJ, Utter AA, Jones MV, Agarwal N, Levin LA (2006). Kinase-dependent differentiation of a retinal ganglion cell precursor.. Invest Ophthalmol Vis Sci.

[26] Yepes M, Sandkvist M, Moore EG, Bugge TH, Strickland DK, Lawrence DA (2003). Tissue-type plasminogen activator induces opening of the blood-brain barrier via the LDL receptor-related protein.. J Clin Invest.

[27] Benchenane K, Berezowski V, Ali C, Fernández-Monreal M, López-Atalaya JP, Brillault J, Chuquet J, Nouvelot A, MacKenzie ET, Bu G, Cecchelli R, Touzani O, Vivien D (2005). Tissue-type plasminogen activator crosses the intact blood-brain barrier by low-density lipoprotein receptor-related protein-mediated transcytosis.. Circulation.

[28] Zhang X, Polavarapu R, She H, Mao Z, Yepes M (2007). Tissue-type plasminogen activator and the low-density lipoprotein receptor-related protein mediate cerebral ischemia-induced nuclear factor-kappaB pathway activation.. Am J Pathol.

[29] Polavarapu R, Gongora MC, Yi H, Ranganthan S, Lawrence DA, Strickland D, Yepes M (2007). Tissue-type plasminogen activator-mediated shedding of astrocytic low-density lipoprotein receptor-related protein increases the permeability of the neurovascular unit.. Blood.

[30] Herz J, Clouthier DE, Hammer RE (1992). LDL receptor-related protein internalizes and degrades uPA-PAI-1 complexes and is essential for embryo implantation.. Cell.

[31] Strickland DK, Gonias SL, Argraves WS (2002). Diverse roles for the LDL receptor family.. Trends Endocrinol Metab.

[32] Campana WM, Li X, Dragojlovic N, Janes J, Gaultier A, Gonias SL (2006). The low-density lipoprotein receptor-related protein is a pro-survival receptor in Schwann cells: possible implications in peripheral nerve injury.. J Neurosci.

[33] Li Y, Gonzalez MI, Meinkoth JL, Field J, Kazanietz MG, Tennekoon GI (2003). Lysophosphatidic acid promotes survival and differentiation of rat Schwann cells.. J Biol Chem.

[34] Lutz C, Nimpf J, Jenny M, Boecklinger K, Enzinger C, Utermann G, Baier-Bitterlich G, Baier G (2002). Evidence of functional modulation of the MEKK/JNK/cJun signaling cascade by the low density lipoprotein receptor-related protein (LRP).. J Biol Chem.

[35] Hardy MM, Feder J, Wolfe RA, Bu G (1997). Low density lipoprotein receptor-related protein modulates the expression of tissue-type plasminogen activator in human colon fibroblasts.. J Biol Chem.

[36] Bu G, Schwartz AL (1998). RAP, a novel type of ER chaperone.. Trends Cell Biol.

[37] Zhuo M, Holtzman DM, Li Y, Osaka H, DeMaro J, Jacquin M, Bu G (2000). Role of tissue plasminogen activator receptor LRP in hippocampal long-term potentiation.. J Neurosci.

[38] Biessen EA, van Teijlingen M, Vietsch H, Barrett-Bergshoeff MM, Bijsterbosch MK, Rijken DC, van Berkel TJ, Kuiper J (1997). Antagonists of the mannose receptor and the LDL receptor-related protein dramatically delay the clearance of tissue plasminogen activator.. Circulation.

[39] Bu G, Warshawsky I, Schwartz AL (1994). Cellular receptors for the plasminogen activators.. Blood.

[40] Zilberberg A, Yaniv A, Gazit A (2004). The low density lipoprotein receptor-1, LRP1, interacts with the human frizzled-1 (HFz1) and down-regulates the canonical Wnt signaling pathway.. J Biol Chem.

[41] Cordeiro MF, Guo L, Luong V, Harding G, Wang W, Jones HE, Moss SE, Sillito AM, Fitzke FW (2004). Real-time imaging of single nerve cell apoptosis in retinal neurodegeneration.. Proc Natl Acad Sci USA.

[42] Guo L, Salt TE, Maass A, Luong V, Moss SE, Fitzke FW, Cordeiro MF (2006). Assessment of neuroprotective effects of glutamate modulation on glaucoma-related retinal ganglion cell apoptosis in vivo.. Invest Ophthalmol Vis Sci.

